# Thyroid Function, Inflammation, and HDL-Cholesterol in Women with Acne: A Real-World Cross-Sectional Study Integrating Biochemistry and Thyroid Ultrasound

**DOI:** 10.3390/jcm15051768

**Published:** 2026-02-26

**Authors:** Maria Madalina Singer, Ștefănița Bianca Vintilescu, Denisa Floriana Vasilica Pirscoveanu, Virginia Maria Rădulescu, Andreea Gabriela Mocanu, Oana-Elena Nicolaescu, Renata Maria Varut, Denisa Preoteasa, Mioara Desdemona Stepan, Ion Dorin Pluta, Cristina Elena Singer

**Affiliations:** 1 Dermatology Department, Central Military Hospital, 010825 Bucharest, Romania; maria.singer1987@gmail.com; 2 Department of Mother and Baby, University of Medicine and Pharmacy of Craiova, 200349 Craiova, Romania; bianca.vintilescu@umfcv.ro (Ș.B.V.); desdemona.stepan@umfcv.ro (M.D.S.); cristina.singer@umfcv.ro (C.E.S.); 3 Department of Neurology, Faculty of Medicine, University of Medicine and Pharmacy of Craiova, 200349 Craiova, Romania; denisa.pirscoveanu@umfcv.ro; 4 Department of Statistics, University of Medicine and Pharmacy of Craiova, 200349 Craiova, Romania; 5 Department of Pharmaceutical Technology, University of Medicine and Pharmacy of Craiova, 200349 Craiova, Romania; oana.nicolaescu@umfcv.ro; 6 Research Methodology Department, Faculty of Pharmacy, University of Medicine and Pharmacy of Craiova, 200349 Craiova, Romania; renata.varut@umfcv.ro; 7 Vâlcea County Emergency Hospital, 240595 Râmnicu Vâlcea, Romania; denisa_chiosa@yahoo.com; 8 Faculty of Medical and Behavioral Sciences, Constantin Brâncuși University of Târgu Jiu, 210185 Târgu Jiu, Romania; dorin.pluta@e-ucb.ro

**Keywords:** acne, thyroid-stimulating hormone, free thyroxine, HDL-cholesterol, neutrophil-to-lymphocyte ratio, thyroid ultrasound, endocrine markers, inflammation

## Abstract

**Background:** Acne in adult women is increasingly recognized as a condition with systemic endocrine–metabolic correlates. Evidence linking acne to thyroid-related abnormalities and cardiometabolic risk markers remains mixed, and integrated real-world evaluations combining thyroid biochemistry, ultrasound metrics, inflammatory indices, and lipid profile are limited. **Methods:** We performed a cross-sectional observational analysis of 80 women with acne who underwent routine laboratory testing and thyroid ultrasound assessment. Thyroid status was defined using TSH (reference 0.4–4.5 mIU/L) and free T4 (0.8–1.8 ng/dL), with an additional TSH-only sensitivity definition (high TSH >4.5 mIU/L). Low HDL-cholesterol (HDL-C) was defined as <50 mg/dL. Group comparisons used Mann–Whitney U tests with Hodges–Lehmann shifts; associations were summarized using odds ratios (ORs) with Fisher’s exact tests; correlations used Spearman’s ρ (TSH log-transformed for correlation analyses) with confidence intervals. Multiple testing was controlled within panels using Benjamini–Hochberg FDR. Analyses were complete-case per comparison. **Results:** Thyroid dysfunction and metabolic–inflammatory abnormalities were common in this cohort. Low HDL-C was more frequent in thyroid dysfunction, and in the TSH-only sensitivity analysis, high TSH (>4.5 mIU/L) was strongly associated with low HDL-C (OR 13.13, 95% CI 1.48–116.04; *p* = 0.020). In a minimal adjusted model including NLR, high TSH remained associated with low HDL-C (adjusted OR 12.93, 95% CI 1.44–115.70; *p* = 0.022). HDL-C showed an inverse association with NLR (ρ = −0.28; *p* = 0.023). Endocrine profiling suggested a positive association between ACTH and log(TSH) (ρ = 0.62; *p* = 0.004), although this did not remain significant after FDR correction. Thyroid ultrasound metrics showed limited correspondence with thyroid biochemistry. **Conclusions:** In women with acne, elevated TSH is associated with substantially higher odds of low HDL-C, independent of inflammatory burden as proxied by NLR, while thyroid ultrasound morphology contributes limited functional information. These findings support integrated thyroid–metabolic assessment in adult female acne and motivate prospective studies incorporating acne severity measures and standardized testing to clarify clinical implications.

## 1. Introduction

Acne vulgaris is a common inflammatory disorder of the pilosebaceous unit, yet in many patients—particularly adult women—it behaves as a chronic condition with meaningful endocrine and metabolic context rather than a purely cutaneous problem. Contemporary overviews of adult female acne emphasize its distinct triggers, persistence, and broader impact, supporting a more integrative clinical assessment beyond lesion-directed management alone [1,2].

From an endocrine perspective, adult female acne is frequently discussed within the spectrum of androgen-related disorders, and expert guidance recommends structured biochemical assessment of androgens in women with adult acne, particularly when clinical features suggest hyperandrogenism or when acne is treatment-resistant [3]. At the same time, acne pathophysiology is not reducible to hormones: inflammatory signaling, innate immune activation, and systemic correlates can meaningfully coexist with (or occur independently of) androgen excess, which helps explain the heterogeneity seen in routine care [2,3].

The thyroid axis has emerged as a plausible—but still incompletely characterized—component of this broader systemic landscape. Small clinical studies have reported associations between acne and markers suggestive of thyroid autoimmunity (e.g., anti-thyroglobulin antibodies), raising the possibility that immune–endocrine coupling may be relevant in a subset of patients [4,5]. In contrast, other investigations have focused on thyroid function tests and related metabolic features, with some reporting lipid profile differences (including HDL-C patterns) without clear separation in standard thyroid hormone measures, underscoring that the direction and clinical meaning of thyroid–acne links may depend on phenotype, sampling, and the set of biomarkers measured [6].

Metabolic risk markers also deserve attention because acne has been repeatedly examined in relation to serum lipids, with systematic synthesis suggesting that lipid differences can be present at the group level, although results vary across settings and designs [7]. In this context, “low HDL-C” is clinically interpretable and may serve as a pragmatic risk signal that can be tested against endocrine strata (including thyroid status) within real-world datasets.

In parallel, there is growing interest in simple hematologic indices as proxies for systemic inflammatory tone in acne. Large-scale observational work has linked elevated hematologic ratios (including NLR) to acne severity, while smaller clinical studies also report shifts in complete blood count-derived markers, supporting the concept that acne-related inflammation may have measurable systemic fingerprints—even if effect sizes are modest and not fully consistent across cohorts [8,9].

Against this background, the remaining gap is not the lack of “single-axis” studies, but the relative scarcity of integrated, clinic-realistic evaluations that consider thyroid biochemistry, thyroid ultrasound morphology, metabolic markers (including HDL-C), and inflammatory indices in the same patient set—reported with effect sizes and uncertainty rather than *p*-values alone. Recent work continues to explore thyroid disorders and acne severity at a population level, reinforcing topical relevance, while also highlighting how much context (definitions, ranges, and measurement framework) shapes inference [9].

Therefore, in a cohort of women diagnosed with acne who underwent multidimensional laboratory assessment and thyroid ultrasound evaluation, this study aimed to (i) examine associations between thyroid dysfunction categories and acne-relevant endocrine markers where available; (ii) quantify relationships between thyroid ultrasound metrics and thyroid function (TSH/FT4); and (iii) evaluate whether low HDL-C is associated with thyroid status, reporting effect sizes with confidence intervals to support clinically readable interpretation.

## 2. Materials and Methods

### 2.1. Study Design and Participants

The study population consisted of 80 female patients with a documented clinical diagnosis of acne vulgaris, evaluated at the Dermatology Department of Craiova Hospital, Romania, and affiliated outpatient services, during the period April 2023 to March 2025. The diagnosis of acne was established by a board-certified dermatologist based on clinical examination and medical records. Cohort assembly and data provenance were based on a retrospective query of routine clinical records, linked laboratory reports, and thyroid ultrasound archives for the study period. We identified all adult women with clinician-documented acne vulgaris who had study-relevant laboratory data and/or a thyroid ultrasound report recorded within the participating service(s). All eligible records meeting these criteria were included without additional sampling. Because the dataset was extracted from anonymized routine-care documentation, the number of patients approached, declining investigations, or otherwise not captured in the laboratory/ultrasound archives could not be reconstructed; therefore, we address potential selection effects by reporting analysis-specific denominators and data availability across domains in the Section 3.

Eligibility criteria included: (i) female sex; (ii) age ≥18 years; (iii) documented diagnosis of acne vulgaris in the medical record; and (iv) availability of at least one laboratory parameter or thyroid ultrasound examination relevant to the study objectives. Patients were not excluded based on acne severity, duration of disease, or current dermatologic treatment, reflecting the real-world clinical setting. Standardized acne severity grading was not routinely documented in the source records and was therefore unavailable for severity-stratified analyses or adjustment. Similarly, acne distribution (facial vs. truncal), disease duration, and a standardized record of current acne treatments at the time of laboratory testing (e.g., systemic antibiotics, isotretinoin, or hormonal therapy) were not consistently captured in the routine documentation. Therefore, we could not stratify analyses by these clinical descriptors or restrict analyses to specific treatment–exposure subgroups.

Because laboratory investigations and thyroid ultrasound examinations were ordered according to clinical judgment rather than a standardized research protocol, the availability of biochemical, endocrine, inflammatory, and ultrasound variables varied across participants. Consequently, analyses were conducted using complete-case data for each specific comparison, and the effective sample size (denominator) is reported for all analyses. No active intervention, randomization, or experimental manipulation was performed. The study did not include a non-acne control group, and all findings represent within-cohort associations. The cross-sectional design precludes causal inference, and observed associations should be interpreted as exploratory and hypothesis-generating. Due to the retrospective observational nature of the study and the use of anonymized clinical data, the requirement for informed consent was waived by the ethics committee, in accordance with national regulations and institutional policies.

### 2.2. Data Sources and Variables

Data were extracted from routine clinical records and entered into a structured database capturing thyroid-related measures, metabolic and inflammatory markers, and endocrine parameters where available. Thyroid function was characterized using serum TSH and free thyroxine (free T4), while thyroid autoimmunity (anti-thyroid peroxidase and anti-thyroglobulin antibodies) was recorded when clinically requested. Metabolic profiling included HDL-cholesterol, LDL-cholesterol, total cholesterol, triglycerides, and fasting glucose. Although glucose was recorded as “fasting glucose” in the laboratory reports, fasting status for other assays and the exact time of sampling were not consistently documented across participants. Accordingly, residual diurnal and postprandial variability—particularly for TSH and lipid measures—cannot be excluded in this real-world cross-sectional dataset. Systemic inflammation was assessed using white blood cell count and differential values; the neutrophil-to-lymphocyte ratio (NLR) was calculated from the recorded neutrophil and lymphocyte percentages. Additional endocrine markers (e.g., prolactin, ACTH, cortisol, gonadotropins, and sex steroids) were included when measured in the clinical workflow, acknowledging that availability varied across individuals and markers.

Variables such as body mass index, smoking status, diet and physical activity indicators, menstrual cycle timing, and structured medication exposure (e.g., isotretinoin, combined oral contraceptives, lipid-lowering agents, or thyroid-active therapy) were not consistently available in the underlying routine-care documentation in a format suitable for adjustment; therefore, these factors could not be included as covariates in multivariable models and are considered sources of residual confounding. In particular, exposure to isotretinoin and combined oral contraceptives could not be reconstructed reliably from the available records; accordingly, no treatment-stratified or treatment-restricted analyses were performed.

**Figure 1:**
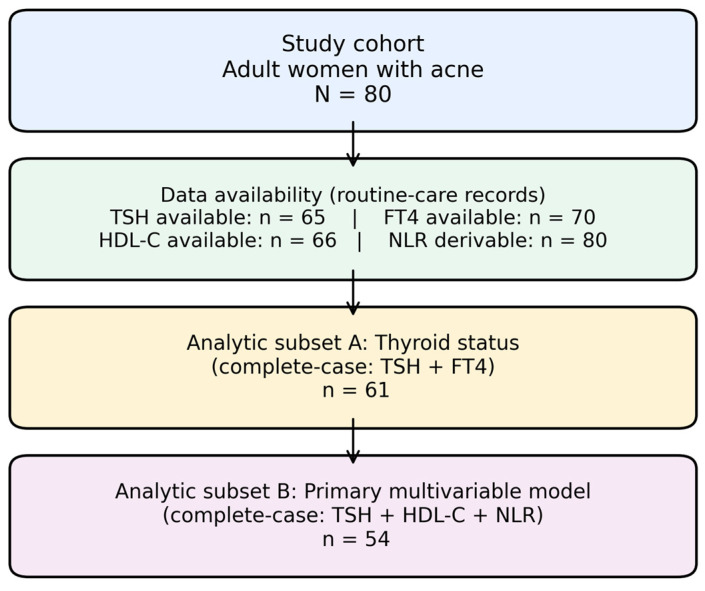
Data availability and analytic flow. A flow schematic summarizing the full cohort (N = 80) and availability of key measurements used in domain-specific analyses, including the thyroid status subset requiring both TSH and FT4 (n = 61) and the complete-case sample used for the primary multivariable logistic regression evaluating high TSH (>4.5 mIU/L) in relation to low HDL-C (<50 mg/dL) with adjustment for NLR (n = 54).

Data availability across domains and the analytic flow from the full cohort to key complete-case subsets are summarized in Figure 1.

### 2.3. Thyroid Ultrasound Acquisition and Derived Measures

Thyroid ultrasound examinations were performed as part of routine clinical care, and archived reports were retrieved from the institutional ultrasound record. Because acquisition parameters (e.g., ultrasound system model, transducer frequency, patient positioning) and standardized reporting frameworks (e.g., TI-RADS or prespecified parenchymal grading) were not consistently documented in the archived reports, these items could not be harmonized retrospectively. Therefore, ultrasound variables were limited to those explicitly reported and could be extracted reproducibly across records. In addition to lobe dimensions and isthmus thickness, qualitative descriptors were extracted when present, including echostructure (e.g., normal vs. abnormal) and vascularity, as recorded in the report. Because these qualitative items were not documented using a standardized grading system, they were summarized descriptively and were not used to derive additional severity categories. When numeric dimensions were available, left and right lobe volumes were derived from lobe length, width, and anteroposterior diameter using the standard ellipsoid formula with the Brunn correction factor (0.479):

Vlobe = L × W × AP × 0.479

Dimensions recorded in millimeters were converted to milliliters assuming 1 mL = 1000 mm^3^. Total thyroid volume was defined as the sum of left and right lobe volumes. Isthmus thickness was analyzed as recorded (mm).

Importantly, if the ultrasound report contained qualitative statements such as “normal” without numeric measurements, the entry was retained as a text-only normal flag and no quantitative dimensions or volumes were imputed for these cases.

### 2.4. Data Cleaning and Harmonization

Before analysis, we applied prespecified, rule-based preprocessing to harmonize coding, units, and interpretability across routine-care records. Laboratory entries recorded as “0” were treated as missing because this code was used as a placeholder for unavailable results rather than a physiologic value. For thyroid ultrasound, when the report contained a qualitative statement indicating a normal examination (e.g., “normal values”) but did not provide quantitative measurements, we retained this information as a narrative “normal ultrasound” flag and left dimension/volume fields missing because imputing structural measurements would not be valid. For fasting glucose and potassium, we performed unit/decimal audits against standard clinical plausibility and the laboratory reference intervals (reported in Table 1). When values followed a recognizable pattern compatible with unit/decimal transcription (e.g., glucose recorded in a mmol/L × 10 format), we applied a deterministic conversion to mg/dL to preserve interpretability; for example, a recorded glucose value of 45 was interpreted as 4.5 mmol/L × 10 and harmonized to 81 mg/dL by multiplication with 1.8. For potassium, values below a physiologically plausible threshold suggestive of a missing decimal point were multiplied by 10, and residual values outside a conservative plausibility range were set to missing. Absolute WBC values showed clustering and reporting heterogeneity across records; therefore, WBC-based classifications were reported descriptively but were not used as inferential predictors. Differential-derived indices (e.g., NLR) were computed only when the underlying differential counts were interpretable, and any clearly implausible differential profile was treated as missing for index derivation. As a prespecified sensitivity check, key estimates were re-calculated after excluding records requiring unit/decimal harmonization and after excluding records flagged for hematology reporting heterogeneity, to confirm that the main inferences were not driven by these preprocessing steps. Unit plausibility audits were performed against the reference intervals reported by the issuing laboratories and the expected clinical units for each assay. For variables where distributions suggested potential unit/decimal inconsistencies (notably fasting glucose and potassium), we applied deterministic, prespecified conversions as described above and retained a flag indicating whether a value required harmonization. To assess robustness, all primary inferential models were re-run after excluding records that required unit/decimal harmonization, and estimates were compared with the main analyses. For hematology, absolute WBC values showed clustering consistent with heterogeneous reporting across sources; therefore, WBC-based classifications were treated as descriptive only, while differential-derived indices (e.g., NLR) were computed only when the underlying differential profile was physiologically plausible. A sensitivity analysis additionally excluded records flagged for hematology reporting heterogeneity to confirm that key associations were not driven by such entries.

### 2.5. Definitions of Thyroid Status and Clinical Thresholds

Reference ranges and clinical thresholds were specified a priori and aligned with the results. Thyroid function was interpreted using TSH and free T4, with a TSH reference range of 0.4–4.5 mIU/L (upper limit 4.5) and a free T4 reference range of 0.8–1.8 ng/dL. Single-marker categories were defined as low TSH (<0.4 mIU/L), high TSH (>4.5 mIU/L), low free T4 (<0.8 ng/dL), and high free T4 (>1.8 ng/dL). When both TSH and free T4 were available, combined thyroid status was classified as euthyroid (TSH 0.4–4.5 and free T4 0.8–1.8), subclinical hypothyroidism (TSH > 4.5 with normal free T4), overt hypothyroidism (TSH > 4.5 with low free T4), subclinical hyperthyroidism (TSH < 0.4 with normal free T4), and overt hyperthyroidism (TSH < 0.4 with high free T4). “Any thyroid dysfunction” was defined as any non-euthyroid combined pattern.

For cardiometabolic and inflammatory profiling, low HDL-cholesterol was defined as <50 mg/dL, and lipid abnormalities were flagged as total cholesterol ≥200 mg/dL, LDL-cholesterol ≥130 mg/dL, and triglycerides ≥150 mg/dL. Hypoglycemia was defined as fasting glucose <70 mg/dL. Leukocytosis was defined as WBC > 10 × 10^3^/µL. NLR was computed as neutrophils (%) divided by lymphocytes (%), and an NLR > 2.0 was used as an elevated threshold. Thyroid autoimmunity positivity was defined using anti-TPO > 35 IU/mL and anti-thyroglobulin >40 IU/mL, while enlarged thyroid volume in women was defined as >18 mL. For liver enzymes, elevated ALT and AST were flagged using thresholds of >45 U/L and >43 U/L, respectively. Prolactin values were extracted from routine laboratory reports as recorded in the dataset. Because unit annotation and laboratory-specific reference intervals were not consistently retained in this retrospective dataset, prolactin-related analyses are interpreted descriptively and treated as exploratory rather than used to support pooled clinical cut-offs. When a binary flag was reported, the threshold reflected the most commonly used upper reference limit in the source reports, and was used for descriptive stratification only.

### 2.6. Statistical Analysis

The initial dataset was compiled and verified using Microsoft Excel 365 (Microsoft Corp., Redmond, WA, USA), where variables were coded, checked for internal consistency, and structured for analysis. Statistical analyses were performed using IBM SPSS Statistics, version 26 (IBM Corp., Armonk, NY, USA). Categorical variables were summarized as n/N (%), using non-missing values as denominators; key prevalence estimates were accompanied by Wilson 95% confidence intervals. Continuous variables were described primarily using median (IQR) and min–max, reflecting skewed distributions across several markers, and the number of observations contributing to each analysis is reported in the corresponding tables.

Inferential analyses were organized according to the study objectives. Associations between thyroid status and low HDL-C (<50 mg/dL) were evaluated using 2 × 2 contingency analyses, reported as odds ratios (ORs) with 95% confidence intervals and two-sided Fisher’s exact tests. In addition to the combined thyroid classification based on TSH and free T4, a TSH-only sensitivity definition (high TSH > 4.5 mIU/L) was examined. Relationships between NLR and HDL-C and between endocrine or ultrasound metrics and thyroid function were assessed using Spearman correlations (ρ), with TSH analyzed on the log scale for correlation analyses. Between-group comparisons of continuous markers by thyroid status were performed using two-sided Mann–Whitney U tests, reported with the Hodges–Lehmann median shift and rank-biserial correlation as effect size. To limit false-positive findings in multi-marker panels, Benjamini–Hochberg false discovery rate (BH–FDR) adjustment was applied within each panel, and both *p*-values and q-values are presented. Confidence intervals for correlations and LOWESS confidence bands were obtained using nonparametric bootstrap procedures, except for ultrasound correlations where Fisher’s z-transform. All tests were two-sided with a nominal significance threshold of *p* < 0.05, and missing data were handled by complete-case analysis for each comparison. Prespecified sensitivity checks repeated the primary models after excluding records requiring unit/decimal harmonization and after excluding records flagged for hematology reporting heterogeneity. Given sparse cell counts for elevated TSH, we assessed model influence by leave-one-out refitting and additionally performed a Firth-penalized logistic regression as a sensitivity analysis.

Inflammation was operationalized using the neutrophil-to-lymphocyte ratio (NLR), analyzed both as a continuous variable and as a prespecified binary indicator (NLR > 2.0 vs. ≤2.0). Continuous associations with HDL-C were quantified using Spearman’s rho with 95% confidence intervals, whereas the categorical contrast (NLR > 2.0 vs. ≤2.0) was examined using Mann–Whitney U tests (with Hodges–Lehmann median shifts) and, where appropriate, logistic regression for low HDL-C. Given the real-world laboratory heterogeneity, we additionally conducted sensitivity analyses excluding records flagged during unit/transcription quality checks for WBC-derived metrics to assess the robustness of NLR–HDL-C findings.

Because several endocrine markers were available only in small subsets, we performed post hoc power calculations for correlation analyses using Fisher’s z approximation to contextualize secondary results. For example, with n = 19 (ACTH–TSH subset), the study has ~0.83 power to detect a correlation of |ρ| = 0.62, but only ~0.40 power to detect a moderate association of |ρ| = 0.40 at α = 0.05 (two-sided), implying limited sensitivity for moderate secondary effects. Accordingly, subset-based endocrine associations are interpreted as exploratory.

In addition, a minimal multivariable logistic regression model was fitted to evaluate whether the association between high TSH (>4.5 mIU/L) and low HDL-C (<50 mg/dL) persisted after adjustment for NLR, and a data completeness (selection) check compared baseline and thyroid-related measures between patients with versus without endocrine panels available. As an additional sensitivity analysis addressing metabolic context using available data, the primary logistic regression was repeated with further adjustment for triglycerides (mg/dL).

## 3. Results

### 3.1. Study Sample and Data Availability

The study included 80 women diagnosed with acne who underwent thyroid ultrasound assessment and laboratory testing as part of routine clinical evaluation. The dataset was finalized for analysis after standardized preprocessing to ensure internal consistency and comparability across records. In particular, laboratory entries coded as 0 were handled as non-informative placeholders and treated as missing values, and ultrasound reports described qualitatively as “normal” without numeric measurements were retained as narrative entries rather than converted into quantitative metrics.

Data completeness varied across domains. Thyroid function tests were available for most participants (TSH: 65/80; free T4: 70/80), with combined thyroid status (TSH and free T4 available simultaneously) definable in 61/80. Metabolic profiling included HDL-C in 66/80 and glucose values in 66/80. Ultrasound-derived total thyroid volume could be computed in 60/80, while 20/80 had ultrasound documented as “normal” in narrative form only. Thyroid autoantibodies were measured in a smaller subset (anti-TPO: 31/80; anti-thyroglobulin: 31/80). Inflammatory markers were broadly available (WBC: 77/80), and the neutrophil-to-lymphocyte ratio (NLR) could be derived across the cohort. Thyroid autoantibodies were available in a subset of participants and included both anti-thyroglobulin and anti-thyroid peroxidase antibodies (Table 1). Analyses were performed using complete-case data per comparison; therefore, analytic sample sizes differ between tests and are reported alongside each estimate. Unit/decimal harmonization and hematology heterogeneity flags were treated as data-quality features; sensitivity checks excluding flagged records were consistent with the main interpretation.

### 3.2. Baseline Biochemical and Endocrine Profile

Baseline distributions are summarized in Table 1. Median TSH was 2.01 mIU/L (IQR 1.30–3.20; n = 65) and median free T4 was 1.08 ng/dL (0.92–1.17; n = 70). Thyroid ultrasound measurements were generally within expected ranges, with a median total thyroid volume of 4.52 mL (4.07–6.47; n = 60) and isthmus thickness of 4.0 mm (4.0–4.0; n = 66).

Among endocrine markers, prolactin values were right-skewed (median 33.1 mIU/mL [14.6–186.8]; n = 44), while ACTH showed a median of 18.0 pg/mL (7.6–30.8; n = 22) in the available subset (Table 1).

The lipid profile showed a median HDL-C of 54.0 mg/dL (44.2–58.0; n = 66), with most total cholesterol, LDL-C, and triglyceride values below conventional thresholds (Table 1). Fasting glucose values were harmonized as described in Section 2 and had a median of 113.4 mg/dL (100.8–115.2; n = 66). Electrolytes and renal markers, when available, were largely within physiologic ranges; after data cleaning, potassium had a median of 4.55 mmol/L (4.20–5.60; n = 57).

### 3.3. Prevalence of Thyroid, Inflammatory, Endocrine, and Metabolic Abnormalities

Prevalence estimates are summarized in Table 2. Using TSH 0.4–4.5 mIU/L and free T4 0.8–1.8 ng/dL, any thyroid dysfunction was identified in 16/61 patients (27.9%), including high TSH in 8/65 (12.3%) and high free T4 in 3/70 (4.3%). Thyroid autoantibody positivity was observed in 3/31 (9.7%) for anti-TPO and 6/31 (19.4%) for anti-thyroglobulin.

Low HDL-C was common (26/66, 39.4%). Prolactin exceeded the applied threshold (>25, as recorded) in 26/44 (59.1%), and NLR > 2.0 was present in 30/79 (38.0%). After glucose harmonization, impaired fasting glucose (≥100 mg/dL) was frequent (54/66, 81.8%), whereas hypoglycemia (<70 mg/dL) was rare (1/66, 1.5%). WBC-based leukocytosis was very common (76/77), but this estimate is treated cautiously due to potential reporting heterogeneity (Table 2, †). Text-only “normal” ultrasound documentation was present in 20/80 (25.0%). In the routine ultrasound records, qualitative descriptors were predominantly reassuring: echostructure and vascularity were reported as normal in all cases (80/80), noting that these are narrative fields without standardized grading, and therefore were summarized descriptively.

### 3.4. Inferential Analyses

#### 3.4.1. Low HDL-C in Relation to Thyroid Status

Low HDL-C (<50 mg/dL) was examined in relation to thyroid status using both the combined thyroid classification (TSH + free T4) and a TSH-only sensitivity definition (TSH ULN = 4.5 mIU/L). In the combined-status analysis (n = 51 with both thyroid classification and HDL-C available), low HDL-C was more frequent in the thyroid dysfunction group (9/16; 56.2%) than in euthyroid participants (13/35; 37.1%), corresponding to an odds ratio (OR) of 2.18 (95% CI 0.65–7.24; Fisher’s exact *p* = 0.235). Although the point estimate suggested higher odds of low HDL-C in thyroid dysfunction, the confidence interval remained compatible with no association in this sample.

In the TSH-only sensitivity analysis (n = 54 with TSH and HDL-C available), low HDL-C was markedly more prevalent among participants with high TSH (>4.5 mIU/L) (7/8; 87.5%) compared with those without high TSH (16/46; 34.8%), yielding an OR of 13.13 (95% CI 1.48–116.27; Fisher’s exact *p* = 0.007). Using an alternative definition of high TSH as >4.0 mIU/L attenuated the association but remained directionally consistent (OR 4.00, 95% CI 1.13–14.15; *p* = 0.035), whereas applying a >5.0 mIU/L cut-off yielded identical classification to >4.5 mIU/L in this dataset and therefore an unchanged odds ratio. The wide confidence interval reflects the small number of patients with high TSH, but the direction and magnitude of the association were consistent across prevalence and odds-based summaries. On the continuous scale, HDL-C showed a weak inverse monotonic association with log-transformed TSH (Spearman ρ = −0.18, 95% CI −0.47 to 0.14; n = 54; *p* = 0.192). Consistent with this pattern, median HDL-C decreased from 54 mg/dL in the lowest log(TSH) quartile to 42 mg/dL in the highest quartile.

**Figure 2:**
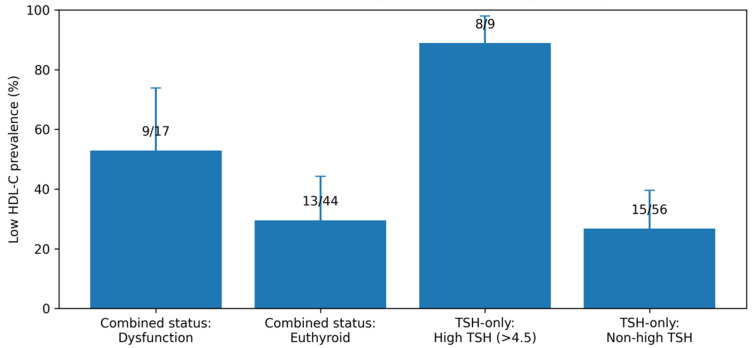
Prevalence of low HDL-C (<50 mg/dL) by thyroid status. Bars show group-specific prevalence with Wilson 95% confidence intervals. The first comparison uses combined thyroid status defined by TSH and free T4 (TSH ULN 4.5 mIU/L), while the second uses a TSH-only sensitivity definition (high TSH > 4.5 mIU/L). Numbers above bars indicate n/N.

For transparency, thyroid biochemistry by HDL strata is summarized. Among participants with both TSH and HDL-C available (n = 54), TSH was higher in the low HDL-C group (median 3.20 [IQR 1.86–6.11] mIU/L; n = 23) than in the normal HDL-C group (median 1.76 [IQR 1.25–2.73] mIU/L; n = 31; Mann–Whitney *p* = 0.020). Among participants with both free T4 and HDL-C available (n = 58), free T4 values were similar between low HDL-C (median 1.08 [IQR 0.92–1.16] ng/dL; n = 23) and normal HDL-C groups (median 1.09 [IQR 0.92–1.21] ng/dL; n = 35; *p* = 0.744) (Figure 2 and Table 3).

Low HDL-C was defined as <50 mg/dL. Group-specific prevalences are reported with Wilson 95% confidence intervals. Odds ratios (ORs) and 95% CIs were computed from 2 × 2 tables; *p*-values are from two-sided Fisher’s exact tests. The combined-status comparison uses a stricter definition (TSH + free T4) and therefore has a smaller analytic sample than the TSH-only sensitivity analysis.

**Figure 3:**
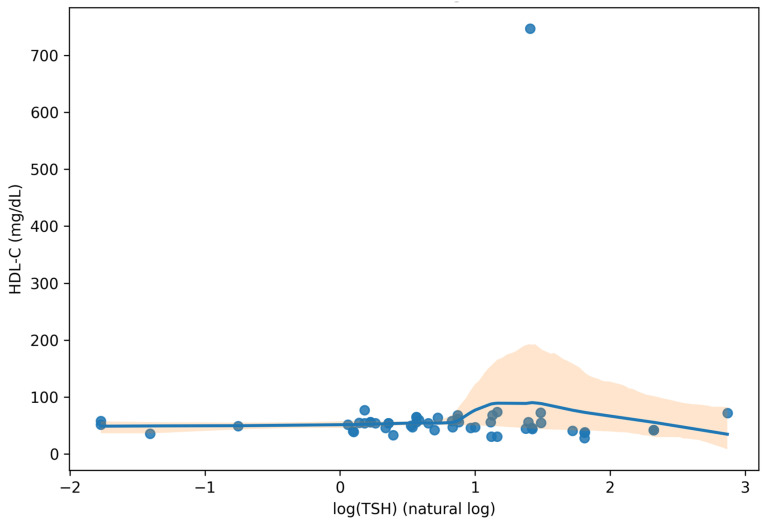
Scatter plot of HDL-C versus log-transformed TSH (natural log). The solid curve represents a LOWESS smoother and the shaded band indicates the 95% confidence interval. Analyses use complete-case data for this panel.

To visualize the dispersion and effect size on the continuous scale, the relationship between HDL-C and log-transformed TSH is shown in Figure 3.

**Figure 4:**
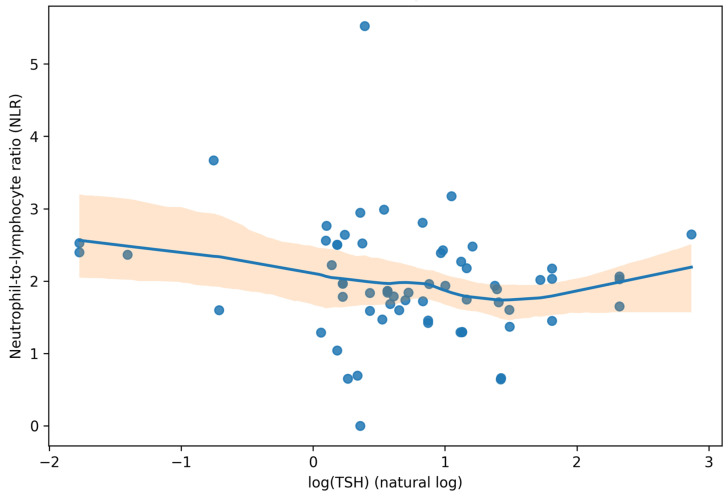
Scatter plot of neutrophil-to-lymphocyte ratio (NLR) versus log-transformed TSH (natural log). The solid curve represents a LOWESS smoother and the shaded band indicates the 95% confidence interval. Analyses use complete-case data for this panel.

For additional context regarding thyroid–inflammation coupling, the relationship between NLR and log-transformed TSH is presented in Figure 4.

#### 3.4.2. Inflammatory–Metabolic Coupling: NLR and HDL-C

**Figure 5:**
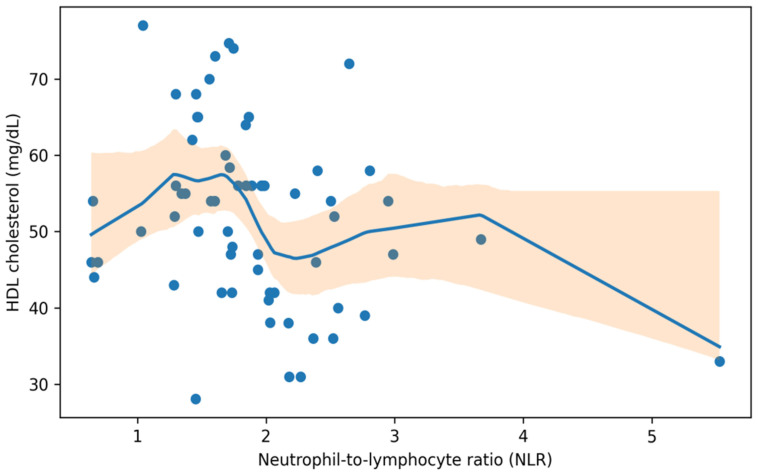
Relationship between the neutrophil-to-lymphocyte ratio (NLR) and HDL-C. Points represent individual participants with both measures available. The solid line shows the LOWESS-smoothed trend, and the shaded band denotes the bootstrap 95% confidence interval.

To explore whether a higher inflammatory burden clustered with an adverse lipid profile, we assessed the association between the neutrophil-to-lymphocyte ratio (NLR) and HDL-C in patients with both measures available. NLR showed a modest inverse correlation with HDL-C (Spearman ρ = −0.28, 95% CI −0.50 to −0.03; n = 65; *p* = 0.023), indicating that higher NLR values tended to co-occur with lower HDL-C levels in this cohort. In contrast, log-transformed TSH was not materially associated with NLR (Spearman ρ = −0.15, 95% CI −0.40 to 0.13; n = 64; *p* = 0.253), suggesting that higher TSH values did not simply track with higher NLR in this dataset. When dichotomized at NLR > 2.0, low HDL-C (<50 mg/dL) was more frequent in the high-NLR stratum (15/22, 68.2%) than in the NLR ≤2.0 stratum (11/43, 25.6%), corresponding to an odds ratio of 6.23 (95% CI 2.02–19.27) and an absolute risk difference of 42.6 percentage points (95% CI 17.1–61.4) in the complete-case subset with both HDL-C and differential counts (n = 65) (Figure 5 and Table 4).

#### 3.4.3. Exploratory Endocrine Markers in Relation to Thyroid Status

**Figure 6:**
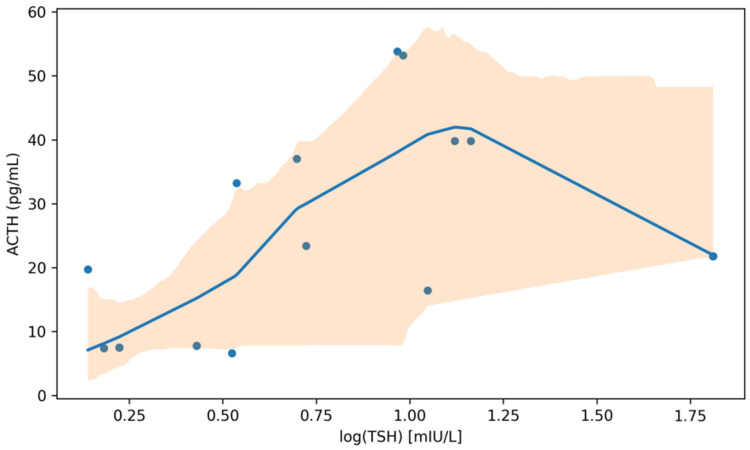
ACTH in relation to TSH (log-transformed). Points represent participants with both measurements available (n = 19). The solid line shows a LOWESS-smoothed trend; the shaded band denotes the bootstrap 95% confidence interval.

Endocrine markers were evaluated in relation to thyroid status using available-case analyses (Table 5). Because endocrine markers were available only in subset analyses and several associations did not remain significant after Benjamini–Hochberg FDR correction, all findings in this subsection are reported as exploratory and hypothesis-generating. In group comparisons (thyroid dysfunction vs. euthyroid), no clear differences were observed across the measured markers, although several comparisons were limited by small subgroup sizes. For example, prolactin values (as recorded) showed overlapping distributions between thyroid dysfunction (median 33.10 [IQR 25.20–183.00], n = 13) and euthyroid patients (median 45.05 [10.07–164.25], n = 24), with a Hodges–Lehmann median shift of 8.35 (95% CI −31.90 to 47.02; *p* = 0.535). FSH tended to be higher in the thyroid dysfunction group (median 15.20 [5.38–15.20], n = 5) than in euthyroid patients (median 6.92 [4.60–10.35], n = 28), but uncertainty remained substantial (*p* = 0.083). This relationship is visualized in Figure 6.

Correlation analyses provided more informative patterns (Table 5). ACTH showed a moderate positive association with log-transformed TSH (Spearman ρ = 0.62, 95% CI 0.42–0.81; n = 19; *p* = 0.004), whereas its association with free T4 was weak and imprecise (ρ = −0.12; *p* = 0.617). However, this association did not remain statistically significant after Benjamini–Hochberg FDR correction (q = 0.063) and is therefore reported as exploratory. Prolactin exhibited a weak positive correlation with log(TSH) (ρ = 0.34, 95% CI 0.09–0.57; n = 39; *p* = 0.033), but this did not persist after multiplicity adjustment within the correlation panel (Table 5). No consistent correlations with free T4 were observed across the available endocrine markers.

#### 3.4.4. Thyroid Ultrasound Metrics in Relation to TSH and Free T4

**Figure 7:**
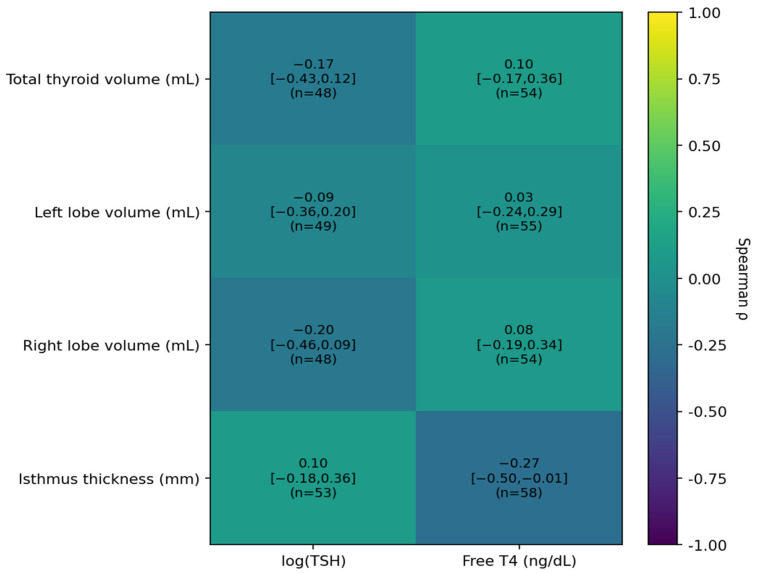
Correlation matrix between thyroid ultrasound metrics and thyroid function. Cells show Spearman correlation coefficients (ρ) with 95% confidence intervals and the available sample size (n) for associations with log(TSH) and free T4 (TSH ULN = 4.5 mIU/L).

Thyroid ultrasound metrics were evaluated against thyroid function using both correlation-based analyses and group comparisons (Table 6; Figure 7). Overall, thyroid volumes showed no meaningful association with thyroid biochemistry. Total thyroid volume correlated weakly with log(TSH) (ρ = −0.17, n = 48, *p* = 0.250) and with free T4 (ρ = 0.10, n = 54, *p* = 0.456), with similar null patterns for left and right lobe volumes (all *p* ≥ 0.168). Qualitative descriptors of parenchymal echostructure and vascularity were uniformly recorded as normal in the available reports (80/80) and therefore were not used to derive a parenchymal score or for inferential associations with TSH/FT4 or antibody status.

For isthmus thickness, the association with log(TSH) was small and non-significant (ρ = 0.10, n = 53, *p* = 0.477). In contrast, isthmus thickness showed a weak inverse correlation with free T4 (ρ = −0.27, n = 58, *p* = 0.039), although this association did not remain significant after multiplicity adjustment within the ultrasound correlation panel (q = 0.312). In group comparisons, ultrasound metrics were broadly comparable between patients with thyroid dysfunction and euthyroid status; median differences were small with wide uncertainty and no evidence of systematic enlargement in the dysfunction group (Table 6, Panel B).

#### 3.4.5. Multivariable Analysis for Low HDL-C

To assess whether the association between elevated TSH and low HDL-C was independent of systemic inflammation, we fitted a minimal multivariable logistic regression model with low HDL-C (<50 mg/dL) as the outcome and high TSH (>4.5 mIU/L) plus NLR as predictors (complete-case, n = 54). High TSH remained associated with markedly higher odds of low HDL-C after adjustment for NLR (adjusted OR 12.93, 95% CI 1.44–115.70; *p* = 0.022), whereas NLR was not independently associated in this model (per +1 unit increase: adjusted OR 1.88, 95% CI 0.85–4.16; *p* = 0.121) (Table 7). In a sensitivity model additionally adjusting for triglycerides (complete-case n = 51), the association between high TSH and low HDL-C remained directionally consistent (adjusted OR 11.63, 95% CI 1.29–104.69; *p* = 0.029). In a sensitivity model additionally including free T4 (complete-case, n = 51), the high-TSH association remained of similar magnitude (adjusted OR 13.86, 95% CI 1.49–129.23; *p* = 0.021), while NLR and free T4 were not independently associated.

#### 3.4.6. Data Completeness Checks for Endocrine Panels

Because endocrine testing was performed in routine care, availability differed by marker (e.g., prolactin measured in 44/80 and ACTH in 22/80 patients). We therefore examined whether patients with endocrine markers available differed systematically from those without. Prolactin availability was associated with a higher frequency of high TSH (18.2% vs. 2.8%; Fisher’s *p* = 0.037), although median TSH and free T4 distributions were similar between groups. ACTH availability was associated with lower HDL-C (median 47.5 vs. 54.5 mg/dL; Mann–Whitney *p* = 0.016), while thyroid function measures showed no meaningful differences. These patterns suggest that endocrine panels were partly driven by clinical suspicion and should be considered when interpreting subset-based endocrine associations.

### 3.5. Integrated Pattern of Associations Across Domains

Across inferential analyses, the most consistent signals linked thyroid status to lipid and endocrine markers, while thyroid ultrasound morphology showed limited coupling with thyroid biochemistry. Using the combined thyroid definition (TSH + free T4), thyroid dysfunction showed a higher prevalence of low HDL-C than euthyroid status, although with wide uncertainty (Table 3; Figure 1). In the TSH-only sensitivity analysis, high TSH (>4.5 mIU/L) was associated with a markedly higher prevalence of low HDL-C (Table 3; Figure 1). In parallel, systemic inflammation—captured by NLR—was inversely associated with HDL-C (Table 4), suggesting that lower HDL-C clustered with higher inflammatory burden in the available subset. Within the endocrine panel, correlations highlighted a moderate positive association between ACTH and log(TSH) (Table 5; Figure 5), whereas most other endocrine markers showed weak or inconsistent relationships with thyroid function and were limited by small sample sizes. Finally, ultrasound-derived thyroid volumes and isthmus thickness showed no strong correlations with log(TSH) or free T4, as summarized in the correlation matrix (Table 6; Figure 7).

In this cohort of women with acne, abnormalities spanning thyroid function, inflammation, and metabolic markers were common (Table 2). Inferential analyses indicated that thyroid status was clinically relevant for lipid risk, with low HDL-C more frequent in patients with thyroid dysfunction and particularly enriched among those with high TSH (>4.5 mIU/L) in the sensitivity analysis (Table 3; Figure 1). In parallel, higher inflammatory burden (NLR) was associated with lower HDL-C, supporting an inflammation–metabolic coupling within the available subset (Table 4). Endocrine profiling suggested a measurable link between the pituitary–thyroid axis and ACTH (Table 5; Figure 6), whereas ultrasound-derived thyroid morphology showed limited correspondence with thyroid biochemistry (Table 6; Figure 7).

## 4. Discussion

Our results support the view that adult female acne may coexist with measurable systemic correlates across endocrine, metabolic, and inflammatory domains. Within this acne cohort, the most clinically interpretable pattern was the enrichment of low HDL-C among patients with elevated TSH (>4.5 mIU/L), which remained evident in a minimal adjusted model accounting for NLR. These findings should be interpreted as within-cohort associations rather than causal determinants of acne, but they may point to a clinically relevant cardiometabolic signal in a subgroup of women presenting with acne. Given the strong influence of adiposity, dietary patterns, and physical activity on HDL-C, the thyroid–HDL association should be interpreted cautiously in the absence of BMI and lifestyle data. Moreover, because no matched non-acne control group was available, we cannot determine whether these thyroid–lipid and inflammation–lipid patterns are specific to acne or reflect broader metabolic trends in the underlying population.

The literature on thyroid function in acne remains mixed. A frequently cited clinical study in post-adolescent acne reported lower HDL-C in acne patients but no clear relationship with thyroid function tests, suggesting that lipid alterations may occur without overt thyroid biochemical separation at the group level. Our findings are consistent with the HDL signal, but extend this perspective by showing that TSH stratification may matter within an acne cohort, identifying a smaller subgroup with markedly higher odds of low HDL-C [10,11,12].

Several case–control studies in adult women have suggested a higher frequency of thyroid autoimmunity markers among acne patients, raising the possibility that immune–endocrine coupling contributes to heterogeneity across cohorts. In our dataset, thyroid antibodies were available only in a subset, limiting inference; however, the prior signal in adult female acne suggests that broader antibody coverage in prospective work may help clarify whether the metabolic–thyroid patterns observed here are enriched in autoimmune phenotypes [13,14].

Inflammatory indices derived from routine blood counts are increasingly studied in acne. Large-scale retrospective evidence has linked elevated hematologic ratios (including NLR) with acne severity, supporting the concept that acne-related inflammation can have systemic “readouts”. In our cohort, the inverse relationship between NLR and HDL-C is compatible with inflammation–lipid coupling, although the absence of acne severity measures prevents testing whether this pattern tracks clinical severity [15,16,17].

In addition, endocrine profiling suggested a moderate positive association between ACTH and log-transformed TSH in the available subset. Although this signal should be considered exploratory due to the limited sample size and the multiple-testing context, it is compatible with physiological coupling between the hypothalamic–pituitary–adrenal (HPA) and hypothalamic–pituitary–thyroid (HPT) axes, where stress-responsive ACTH/cortisol activity may co-occur with adaptive shifts in pituitary–thyroid regulation. In adult acne, neuroendocrine stress pathways have been proposed to interact with inflammatory signaling; therefore, concurrent variation across the HPA and HPT axes may be plausible in a subset of patients and merits prospective confirmation with standardized sampling.

Thyroid ultrasound measurements showed limited correspondence with biochemical thyroid function in our analyses. This “negative” finding is clinically useful: it suggests that routine morphologic metrics (volume, isthmus thickness) may not substitute for functional testing in this setting, and that the signal of interest—where present—may be primarily biochemical rather than structural [18,19].

This study has several important limitations. First, several variables were available only in clinically driven subsets (endocrine panels and antibodies), and complete-case estimation may therefore be affected by informative missingness; in particular, if testing was more frequent in patients perceived as higher risk, subset-based associations may be inflated and should be interpreted as exploratory. Second, participants underwent testing and thyroid ultrasound as part of routine clinical care in a single region, suggesting a cohort already under medical surveillance; consequently, prevalence estimates of thyroid dysfunction and metabolic abnormalities may overestimate rates in unselected adult women with acne and may reflect local practice patterns. Third, laboratory measurement heterogeneity across sources—including unit/decimal transcription issues requiring deterministic harmonization and variability in hematology reporting—may introduce non-differential measurement error that would be expected to attenuate correlation and regression estimates (bias toward the null), although differential misclassification cannot be fully excluded. Fourth, the timing of blood sampling relative to the menstrual cycle and ongoing acne treatments was not standardized or consistently recorded; together with incomplete capture of BMI/adiposity, diet, physical activity, smoking, insulin resistance proxies, and medication exposures (e.g., isotretinoin, combined oral contraceptives, lipid- or thyroid-active agents), residual confounding may either exaggerate or attenuate observed endocrine–lipid associations depending on prescribing and referral patterns. Finally, the cross-sectional design supports within-cohort clustering signals but precludes causal inference or temporal ordering between thyroid status, inflammation, and lipid profiles.

Despite these constraints, the clustering of high TSH with low HDL-C, together with the observed inflammation–lipid coupling, supports a pragmatic, targeted workflow rather than universal screening. In adult women presenting with acne—particularly those with persistent/refractory disease and/or clinical features suggestive of cardiometabolic risk (e.g., prior dyslipidemia, central adiposity, family history of metabolic disease, hypertension, or impaired fasting glucose)—a reasonable first step is biochemical assessment with TSH and FT4 alongside a basic lipid profile. Thyroid ultrasound should be reserved for suspected structural thyroid disease (palpable abnormalities, compressive symptoms) or clinical/laboratory features suggestive of autoimmune thyroiditis, rather than used routinely to triage dyslipidemia risk in acne. Residual confounding by adiposity, lifestyle factors, and medication exposures likely persists and should be considered when interpreting these within-cohort associations. Finally, as AI-enabled tools are increasingly used for pre-consultation triage in dermatology, our endocrine–metabolic findings may represent pragmatic downstream targets within an “AI-to-clinic” pathway. In a recent evaluation of a multimodal large language model (GPT-4o) using clinical images of acne and rosacea, the model achieved high diagnostic performance (overall sensitivity 93.0% [95% CI, 81.4–97.6] and specificity 97.7% [95% CI, 87.9–99.9]). In practice, AI-assisted acne recognition may help prioritize timely clinical review; our data suggest that, among adult women presenting with acne, those with cardiometabolic risk features or persistent disease may particularly benefit from a basic thyroid–lipid assessment (TSH/FT4 and HDL-C) under clinician oversight rather than automated treatment recommendations [19]. Prospective studies with standardized sampling, acne severity scoring, and treatment documentation are needed to validate whether this profile identifies a reproducible subgroup and whether it has implications for long-term metabolic risk stratification.

## 5. Conclusions

In this cohort of women with acne, thyroid-related abnormalities and cross-domain metabolic–inflammatory signals were common. The most consistent finding was a strong association between elevated TSH (>4.5 mIU/L) and low HDL-C, which persisted in a minimal adjusted model accounting for inflammatory burden (NLR). In parallel, HDL-C showed an inverse relationship with NLR, supporting a modest inflammation–lipid coupling within the available subset. Endocrine profiling suggested a measurable link between ACTH and the thyroid axis (ACTH positively correlated with log-transformed TSH), although the limited availability of endocrine panels warrants cautious interpretation.

Routine thyroid ultrasound metrics showed limited correspondence with thyroid biochemistry, indicating that functional variation captured by laboratory testing may be more informative than morphology in this dataset. Overall, these findings support considering integrated thyroid–metabolic assessment in selected adult women with acne—particularly those with treatment-resistant disease, symptoms suggestive of thyroid dysfunction, or cardiometabolic risk features—while prospective studies with standardized phenotyping are needed to clarify clinical utility.

## Figures and Tables

**Table 1:** Baseline characteristics and data availability (N = 80).

**Domain**	**Variable**	**n**	**Missing, n (%)**	**Median [IQR]**	**Min–Max**
Thyroid function					
Thyroid function	TSH (mIU/L)	65	15 (18.8%)	2.01 [1.30–3.20]	0.17–17.60
Thyroid function	Free T4 (ng/dL)	70	10 (12.5%)	1.08 [0.92–1.17]	0.28–3.23
Thyroid autoimmunity					
Thyroid autoimmunity	Anti-TPO antibodies (IU/mL)	31	49 (61.3%)	3.5 [2.3–12.1]	1.5–876.0
Thyroid autoimmunity	Anti-thyroglobulin antibodies (IU/mL)	31	49 (61.3%)	12.6 [5.0–25.6]	0.1–207.0
**Thyroid ultrasound (numeric subset)**					
Thyroid ultrasound	Isthmus thickness (mm)	66	14 (17.5%)	4.0 [4.0–4.0]	3.0–7.0
Thyroid ultrasound	Left lobe volume (mL)	61	19 (23.8%)	2.28 [1.95–3.21]	1.10–7.41
Thyroid ultrasound	Right lobe volume (mL)	60	20 (25.0%)	2.28 [1.95–3.23]	0.48–7.79
Thyroid ultrasound	Total thyroid volume (mL)	60	20 (25.0%)	4.52 [4.07–6.47]	2.50–15.20
Thyroid ultrasound	Ultrasound recorded as “normal” (text-only)	80	0 (0.0%)	20/80 (25.0%)	16.8–35.5%
**Metabolic profile**					
Metabolic profile	Plasma glucose (mg/dL)	66	14 (17.5%)	113.4 [100.8–115.2]	63.0–122.4
Metabolic profile	Total cholesterol (mg/dL)	63	17 (21.2%)	172.0 [160.0–180.0]	99.0–290.0
Metabolic profile	LDL-C (mg/dL)	66	14 (17.5%)	80.0 [71.0–89.0]	21.0–180.0
Metabolic profile	HDL-C (mg/dL)	66	14 (17.5%)	54.0 [44.2–58.0]	28.1–77.0
Metabolic profile	Triglycerides (mg/dL)	65	15 (18.8%)	65.0 [45.0–68.0]	3.0–390.0
Metabolic profile	Non-HDL-C (mg/dL)	62	18 (22.5%)	121.5 [108.5–130.0]	27.0–241.0
Metabolic profile	TG/HDL ratio	63	17 (21.2%)	1.20 [0.82–1.46]	0.07–12.58
**Renal function and electrolytes**					
Renal/electrolytes	Urea (mg/dL)	64	16 (20.0%)	18.0 [18.0–18.0]	15.0–19.0
Renal/electrolytes	Creatinine (mg/dL)	66	14 (17.5%)	0.56 [0.52–0.59]	0.38–0.71
Renal/electrolytes	Uric acid (mg/dL)	32	48 (60.0%)	3.2 [3.2–3.2]	3.0–13.2
Renal/electrolytes	Sodium (mmol/L)	30	50 (62.5%)	137.0 [136.2–138.0]	136.0–148.0
Renal/electrolytes	Potassium (mmol/L)	57	23 (28.7%)	4.55 [4.20–5.60]	3.90–6.10
Renal/electrolytes	Chloride (mmol/L)	30	50 (62.5%)	103.5 [101.2–105.0]	100.0–106.0
Renal/electrolytes	Calcium (mg/dL)	48	32 (40.0%)	9.30 [9.20–9.30]	9.00–9.50
**Liver enzymes**					
Liver enzymes	ALT (U/L)	47	33 (41.2%)	16.0 [15.0–16.0]	12.0–19.0
Liver enzymes	AST (U/L)	46	34 (42.5%)	19.0 [18.0–19.0]	10.0–20.0
**Hematology and inflammation**					
Hematology	Hemoglobin (g/dL)	77	3 (3.8%)	12.9 [12.8–12.9]	12.6–13.7
Hematology	White blood cells (10^3^/µL)	77	3 (3.8%)	18.80 [18.80–18.90]	1.80–39.80
Hematology	Platelets (10^3^/µL)	75	5 (6.2%)	330 [328–376]	165–393
Hematology	Neutrophils (%)	79	1 (1.3%)	60.0 [56.1–64.2]	34.7–80.1
Hematology	Lymphocytes (%)	79	1 (1.3%)	32.5 [27.0–36.3]	14.5–54.4
Hematology	Monocytes (%)	79	1 (1.3%)	6.0 [4.9–7.0]	0.0–10.0
Hematology	Eosinophils (%)	79	1 (1.3%)	1.4 [1.3–1.9]	0.0–11.6
Hematology	Basophils (%)	79	1 (1.3%)	0.5 [0.5–0.6]	0.0–11.0
Hematology	ESR (mm/h)	60	20 (25.0%)	5 [5–6]	4–7
**Exploratory endocrine markers**					
Endocrine	Total testosterone (ng/dL)	19	61 (76.2%)	25.0 [16.8–54.0]	10.0–90.0
Endocrine	LH (mIU/mL)	20	60 (75.0%)	6.15 [4.88–7.39]	3.00–9.50
Endocrine	FSH (mIU/mL)	42	38 (47.5%)	6.18 [4.64–10.50]	0.21–27.00
Endocrine	Prolactin (mIU/mL)	44	36 (45.0%)	33.1 [14.6–186.8]	2.3–473.0
Endocrine	Estradiol (pg/mL)	37	43 (53.8%)	57.3 [28.0–84.6]	0.4–293.9
Endocrine	Progesterone (ng/mL)	10	70 (87.5%)	0.38 [0.24–0.76]	0.10–12.70
Endocrine	Cortisol (ng/dL)	8	72 (90.0%)	8.1 [7.1–9.3]	7.0–17.8
Endocrine	ACTH (pg/mL)	22	58 (72.5%)	18.0 [7.6–30.8]	6.6–53.8
Endocrine	Parathyroid hormone (pg/mL)	34	46 (57.5%)	35.6 [21.7–51.9]	1.0–67.6

**Table 2:** Prevalence of thyroid, inflammatory, endocrine, metabolic, and ultrasound abnormalities (TSH ULN = 4.5 mIU/L).

**Parameter**	**n/N (%)**	**95% CI**	**N Available**
Thyroid status (TSH 0.4–4.5 mIU/L; free T4 0.8–1.8 ng/dL)			
Low TSH (<0.4 mIU/L)	3/65 (4.6%)	1.6–12.7%	65
High TSH (>4.5 mIU/L)	8/65 (12.3%)	6.4–22.5%	65
Low free T4 (<0.8 ng/dL)	5/70 (7.1%)	3.1–15.7%	70
High free T4 (>1.8 ng/dL)	3/70 (4.3%)	1.5–11.9%	70
Subclinical hypothyroidism (TSH > 4.5 with normal FT4)	9/61 (14.8%)	8.1–25.5%	61
Overt hypothyroidism (TSH > 4.5 with low FT4)	0/61 (0.0%)	0.0–5.9%	61
Subclinical hyperthyroidism (TSH < 0.4 with normal FT4)	0/61 (0.0%)	0.0–5.9%	61
Overt hyperthyroidism (TSH < 0.4 with high FT4)	3/61 (4.9%)	1.7–13.5%	61
Any thyroid dysfunction (any non-euthyroid combined pattern)	16/61 (27.9%)	16.8–38.4%	61
**Inflammatory and endocrine markers (subset-dependent)**			
Leukocytosis (WBC > 10 × 10^3^/µL) †	76/77 (98.7%)	93.0–99.8%	77
NLR > 2.0	30/79 (38.0%)	28.1–49.0%	79
Prolactin > 25 (as recorded)	26/44 (59.1%)	44.4–72.4%	44
Metabolic abnormalities			
Low HDL-C (<50 mg/dL)	26/66 (39.4%)	28.5–51.5%	66
Total cholesterol ≥ 200 mg/dL	2/63 (3.2%)	0.9–10.9%	63
LDL-C ≥ 130 mg/dL	1/66 (1.5%)	0.3–8.1%	66
Triglycerides ≥ 150 mg/dL	1/65 (1.5%)	0.3–8.2%	65
Hypoglycemia (glucose < 70 mg/dL) ‡	1/66 (1.5%)	0.3–8.1%	66
Glucose ≥ 100 mg/dL ‡	54/66 (81.8%)	70.9–89.3%	66
Glucose ≥ 126 mg/dL ‡	0/66 (0.0%)	0.0–5.5%	66
**Liver enzymes**			
ALT > 45 U/L	0/47 (0.0%)	0.0–7.6%	47
AST > 43 U/L	0/46 (0.0%)	0.0–7.7%	46
Thyroid antibodies and ultrasound			
Anti-TPO > 35 IU/mL	3/31 (9.7%)	3.3–24.9%	31
Anti-thyroglobulin > 40 IU/mL	6/31 (19.4%)	9.2–36.3%	31
Enlarged thyroid volume (>18 mL, women)	0/60 (0.0%)	0.0–6.0%	60
Ultrasound documented as “normal” (text-only)	20/80 (25.0%)	16.8–35.5%	80

Prevalence is reported as n/N (%) with two-sided 95% Wilson confidence intervals, using non-missing values as denominators (N available is shown per row). Thyroid dysfunction is defined from combined TSH and free T4 as reported in the table. † WBC values showed strong clustering and potential unit/reporting heterogeneity; WBC-derived classifications are reported descriptively and were not used for inferential analyses. ‡ Fasting glucose values were harmonized as described in Section 2 (mmol/L × 10 to mg/dL by multiplying by 1.8).

**Table 3:** Association between thyroid status and low HDL-C (<50 mg/dL) (TSH ULN = 4.5 mIU/L).

Comparison	N (Available)	Low HDL in Group 1	Low HDL in Group 2	OR (95% CI)	*p*-Value (Fisher)
Thyroid dysfunction vs. euthyroid (combined TSH + free T4; TSH ULN 4.5)	51	9/16 (56.2%), 95% CI 33.2–76.9%	13/35 (37.1%), 95% CI 23.2–53.7%	2.18 (0.65–7.24)	0.235
High TSH (>4.5 mIU/L) vs. non-high TSH	54	7/8 (87.5%), 95% CI 56.5–98.0%	16/46 (33.3%), 95% CI 21.4–47.9%	13.30 (1.48–116.27)	0.007

**Table 4:** Inflammatory–metabolic association: NLR and HDL-C.

Association	n	Spearman ρ	95% CI	*p*-Value
NLR vs. HDL-C	65	−0.28	−0.50–−0.03	0.023

NLR, neutrophil-to-lymphocyte ratio (neutrophils %/lymphocytes %). Spearman’s rho is reported with a bootstrap 95% CI.

**Table 5:** Endocrine markers by thyroid status and their associations with thyroid function (TSH ULN = 4.5 mIU/L).

**(A) Group Comparisons (Thyroid Dysfunction vs. Euthyroid)**										
**Marker**	**n (Dysfunction)**	**Median [IQR] (Dysfunction)**	**n (Euthyroid)**	**Median [IQR] (Euthyroid)**	**HL Δ (dys−eu)**	**HL 95% CI**	**Rank-Biserial r**	**r 95% CI**	***p*** **-Value**	**q (BH–FDR)**
Total testosterone (ng/dL)	6	31.50 [17.92–51.75]	7	35.00 [14.75–63.50]	2.75	−37.00–33.75	0.13	−0.56–0.70	0.720	0.751
FSH (mIU/mL)	5	15.20 [5.38–15.20]	28	6.92 [4.60–10.35]	4.95	−0.93–16.50	−0.52	−1.00–0.49	0.083	0.501
Prolactin (as recorded)	13	33.10 [25.20–183.00]	24	45.05 [10.07–164.25]	8.35	−31.90–47.02	−0.13	−0.46–0.27	0.535	0.751
Estradiol (pg/mL)	7	100.00 [23.35–185.55]	17	57.40 [31.70–68.50]	31.50	−35.40–202.50	−0.20	−0.74–0.47	0.465	0.751
Cortisol (ng/dL)	3	9.30 [9.20–9.30]	4	7.05 [7.00–9.78]	2.15	−8.50–2.30	−0.50	−1.00–0.50	0.368	0.751
ACTH (pg/mL)	8	24.45 [14.62–31.00]	11	12.60 [7.18–30.80]	8.80	−15.70–21.40	−0.25	−0.61–0.14	0.225	0.675
**(B) Spearman Correlations with log(TSH) and Free T4**										
**Marker**		**Predictor**	**n**	**Spearman ρ**	**95% CI**		***p*** **-Value**		**q (BH–FDR)**	
Total testosterone (ng/dL)		log(TSH)	16	0.27	−0.26–0.64		0.313		0.626	
		Free T4 (ng/dL)	16	−0.23	−0.65–0.25		0.392		0.669	
LH (mIU/mL)		log(TSH)	17	0.13	−0.29–0.56		0.625		0.729	
		Free T4 (ng/dL)	19	−0.28	−0.65–0.18		0.251		0.586	
FSH (mIU/mL)		log(TSH)	34	0.13	−0.25–0.44		0.478		0.669	
		Free T4 (ng/dL)	28	−0.06	−0.44–0.27		0.756		0.819	
Prolactin (as recorded)		log(TSH)	39	0.34	0.09–0.57		0.033		0.233	
		Free T4 (ng/dL)	42	−0.05	−0.35–0.20		0.737		0.819	
Estradiol (pg/mL)		log(TSH)	11	−0.06	−0.45–0.47		0.873		0.873	
		Free T4 (ng/dL)	11	−0.14	−0.53–0.45		0.708		0.819	
ACTH (pg/mL)		log(TSH)	19	0.62	0.42–0.81		0.004		0.063	
		Free T4 (ng/dL)	19	−0.12	−0.57–0.45		0.617		0.729	
Cortisol (ng/dL)		log(TSH)	7	−0.39	−0.91–0.63		0.389		0.669	
		Free T4 (ng/dL)	7	0.00	−0.75–0.75		1.000		1.000	

Thyroid dysfunction was defined using combined TSH and free T4 categories (TSH reference range 0.4–4.5 mIU/L; free T4 0.8–1.8 ng/dL). Panel A uses two-sided Mann–Whitney U tests with Hodges–Lehmann (HL) median shift (dysfunction−euthyroid) and rank-biserial correlation; 95% CIs are bootstrap-based. Panel B reports Spearman correlations with bootstrap 95% CIs. q-values are Benjamini–Hochberg FDR adjusted within each panel. Prolactin is reported as recorded in the dataset.

**Table 6:** Thyroid ultrasound metrics in relation to thyroid function (TSH ULN = 4.5 mIU/L).

**(A) Spearman Correlations of Ultrasound Metrics with log(TSH) and Free T4.**								
**Ultrasound Metric**	**Predictor**		**n**	**Spearman ρ**	**95% CI**		***p*** **-Value**	**q (BH–FDR)**
Total thyroid volume (mL)	log(TSH)		48	−0.17	−0.43–0.12		0.250	0.655
	Free T4 (ng/dL)		54	0.10	−0.17–0.36		0.456	0.655
Left lobe volume (mL)	log(TSH)		49	−0.09	−0.36–0.20		0.546	0.655
	Free T4 (ng/dL)		55	0.03	−0.24–0.29		0.831	0.831
Right lobe volume (mL)	log(TSH)		48	−0.20	−0.46–0.09		0.168	0.655
	Free T4 (ng/dL)		54	0.08	−0.19–0.34		0.573	0.655
Isthmus thickness (mm)	log(TSH)		53	0.10	−0.18–0.36		0.477	0.655
	Free T4 (ng/dL)		58	−0.27	−0.50–−0.01		0.039	0.312
**(B) Group Comparisons of Ultrasound Metrics (Thyroid Dysfunction vs. Euthyroid).**								
**Ultrasound Metric**	**n (Dysfunction)**	**Median [IQR] (Dysfunction)**	**n (Euthyroid)**	**Median [IQR] (Euthyroid)**	**HL Δ (dys−eu)**	**HL 95% CI**	***p*** **-Value**	**q (BH–FDR)**
Total thyroid volume (mL)	13	4.74 [4.16–7.33]	33	4.49 [4.10–6.43]	0.12	−0.52–1.53	0.788	0.884
Left lobe volume (mL)	13	2.47 [1.95–3.42]	34	2.25 [1.95–3.11]	0.19	−0.13–0.91	0.391	0.782
Right lobe volume (mL)	13	2.33 [2.09–2.74]	33	2.28 [1.95–3.29]	−0.03	−0.45–0.46	0.884	0.884
Isthmus thickness (mm)	14	4.00 [4.00–4.00]	36	4.00 [4.00–4.00]	0.0	0.00–0.00	0.119	0.476

Panel A: Spearman’s rho with approximate 95% CIs computed using Fisher’s z-transform; q-values are BH–FDR adjusted within the panel. Panel B: Two-sided Mann–Whitney U tests with Hodges–Lehmann (HL) median shift (dysfunction−euthyroid); q-values are BH–FDR adjusted within the panel. Ultrasound volumes are derived from standardized lobe dimensions (Brunn ellipsoid factor 0.479; mm converted to mL).

**Table 7:** Multivariable logistic regression for low HDL-C (<50 mg/dL).

Predictor	Adjusted OR (95% CI)	*p*-Value
High TSH (>4.5 mIU/L)	12.93 (1.44–115.70)	0.022
NLR (per +1 unit)	1.88 (0.85–4.16)	0.121

Outcome: Low HDL-C (<50 mg/dL). Model: Logistic regression with predictors high TSH (>4.5 mIU/L) and NLR; complete-case analysis (n = 54). NLR was computed as neutrophil (%) divided by lymphocyte (%).

## Data Availability

Data are contained within the article.

## References

[1] Dréno B., Bagatin E., Blume-Peytavi U., Rocha M., Gollnick H. (2018). Female Type of Adult Acne: Physiological and Psychological Considerations and Management. J. Dtsch. Dermatol. Ges..

[2] Bagatin E., Freitas T.H.P., Rivitti-Machado M.C., Machado M.C.R., Ribeiro B.M., Nunes S. (2019). Adult Female Acne: A Guide to Clinical Practice. Int. J. Womens Dermatol..

[3] Carmina E., Azziz R., Bergfeld W.F., Escobar-Morreale H.F., Futterweit W., Huddleston H.G., Spritzer P.M., Tan M.H., Witchel S.F., Yildiz B.O. (2022). Female Adult Acne and Androgen Excess: A Report from the Multidisciplinary Androgen Excess and PCOS Committee. J. Endocr. Soc..

[4] Vergou T., Mantzou E., Tseke P., Moustou A.E., Katsambas A., Alevizaki M., Antoniou C. (2012). Association of Thyroid Autoimmunity with Acne in Adult Women. J. Eur. Acad. Dermatol. Venereol..

[5] Ekiz O., Balta I., Unlu E., Bulbul Sen B., Rifaioğlu E.N., Dogramaci A.C. (2015). Assessment of thyroid function and lipid profile in patients with postadolescent acne in a Mediterranean population from Turkey. Int. J. Dermatol..

[6] Sobhan M., Seif Rabiei M.A., Amerifar M. (2020). Correlation Between Lipid Profile and Acne Vulgaris. Clin. Cosmet. Investig. Dermatol..

[7] Wiesel V., Weissmann S., Cohen B., Golan-Tripto I., Horev A. (2024). Elevated hematologic ratios are correlated with acne severity: A national, retrospective cohort study. Front. Med..

[8] Chen T., Chen Y., Shao X., Chen J., Liu L., Li Y., Pu Y., Chen J. (2023). Hematological parameters in patients with acnes. J Cosmet Dermatol..

[9] Bungau A.F., Tit D.M., Bungau S.G., Vesa C.M., Radu A.-F., Marin R.C., Endres L.M., Moleriu L.-C. (2024). Exploring the Metabolic and Endocrine Preconditioning Associated with Thyroid Disorders: Risk Assessment and Association with Acne Severity. Int. J. Mol. Sci..

[10] James W.D. (2005). Acne. N. Engl. J. Med..

[11] Niepomniszcze H., Amad R.H. (2001). Skin Disorders and Thyroid Diseases. J. Endocrinol. Investig..

[12] Endres L., Tit D.M., Bungau S., Pascalau N.A., Maghiar Țodan L., Bimbo-Szuhai E., Iancu G.M., Negrut N. (2021). Incidence and Clinical Implications of Autoimmune Thyroiditis in the Development of Acne in Young Patients. Diagnostics.

[13] Nuzzo V., Tauchmanova L., Colasanti P., Zuccoli A., Colao A. (2011). Idiopathic chronic urticaria and thyroid autoimmunity: Experience of a single center. Derm. Endocrinol..

[14] Bucurica S., Nancoff A.S., Dutu M., Mititelu M.R., Gaman L.E., Ioniță-Radu F., Jinga M., Maniu I., Ruța F. (2024). Exploring the Relationship between Lipid Profile, Inflammatory State and 25-OH Vitamin D Serum Levels in Hospitalized Patients. Biomedicines.

[15] Cao N., Li X., Zhang W., Wang Q., Liang Y., Zhou F., Xiao X. (2022). Research progress of signaling pathways of the natural substances intervene dyslipidemia (Review). Exp. Ther. Med..

[16] Ionita-Radu F., Patoni C., Nancoff A.S., Marin F.-S., Gaman L., Bucurica A., Socol C., Jinga M., Dutu M., Bucurica S. (2024). Berberine Effects in Pre-Fibrotic Stages of Non-Alcoholic Fatty Liver Disease—Clinical and Pre-Clinical Overview and Systematic Review of the Literature. Int. J. Mol. Sci..

[17] Petranović Ovčariček P., Giovanella L. (2025). Thyroid Ultrasonography: Much Ado About Nothing? A Provocative Analysis. Cancers.

[18] Carnazza M., Quaranto D., DeSouza N., Moscatello A.L., Garber D., Hemmerdinger S., Islam H.K., Tiwari R.K., Li X.-M., Geliebter J. (2025). The Current Understanding of the Molecular Pathogenesis of Papillary Thyroid Cancer. Int. J. Mol. Sci..

[19] Boostani M., Bánvölgyi A., Goldust M., Cantisani C., Pietkiewicz P., Lőrincz K., Holló P., Wikonkál N.M., Paragh G., Kiss N. (2025). Diagnostic Performance of GPT-4o and Gemini Flash 2.0 in Acne and Rosacea. Int. J. Dermatol..

